# Left ventricular lipoma resected using thoracoscope-assisted limited sternotomy: A case report and literature review

**DOI:** 10.1097/MD.0000000000011436

**Published:** 2018-08-03

**Authors:** Xiangfei Sun, Guangyi Liu, Hwahwi Kim, Wenyu Sun

**Affiliations:** a Department of Cardiovascular Surgery, Qilu Hospital of Shandong University, Qingdao; b Department of Cardiothoracic Surgery, Fangzi District People's Hospital, Weifang Heart Disease Hospital, Weifang, Shandong Province, People's Republic of China; c University of Georgia, Athens, GA.

**Keywords:** cardiac tumor, left ventricular lipoma

## Abstract

**Rationale:**

A cardiac lipoma is an uncommon primary tumor, with a reported incidence ranging from 2.9% to 8% among all benign cardiac tumors. Although the prognosis in most asymptomatic cases is good during longterm follow-up, some reports have shown that untreated cardiac lipomas may be fatal when they cause arrhythmic or obstructive symptoms.

**Patient concerns:**

We present a rare case of left ventricular (LV) lipoma. The mass measured 25 mm 10 mm, with a pedicle on the LV posterior wall near the apex.

**Diagnoses:**

The patient was diagnosed as left ventricular lipoma using echocardiography.

**Interventions:**

The LV lipoma was resected using thoracoscopy-assisted limited sternotomy.

**Outcomes:**

Histopathologic examination was consistent with lipoma. No signs of recurrence were detected on an echocardiogram during a 3-month follow-up period.

**Lessons:**

We performed a comprehensive review of relevant literature and summarized the known 21 cases from 1980 to 2017. LV lipoma may present with or without symptoms, and endoscopic resection may be a good alternative to open surgery.

## 1 Introduction

A cardiac lipoma is an uncommon primary tumor, with a reported incidence ranging from 2.9% to 8% among all benign cardiac tumors.^[1,2]^ Although the prognosis in most asymptomatic cases is good during long-term follow-up, some reports have shown that untreated cardiac lipomas may be fatal when they cause arrhythmic or obstructive symptoms.^[3]^ We present a rare case of left ventricular (LV) lipoma. The mass measured 25 mm × 10 mm, with a pedicle on the LV posterior wall near the apex, and was diagnosed with echocardiography and resected using thoracoscopy-assisted limited sternotomy.

## 2 Case presentation

**Figure 1:**
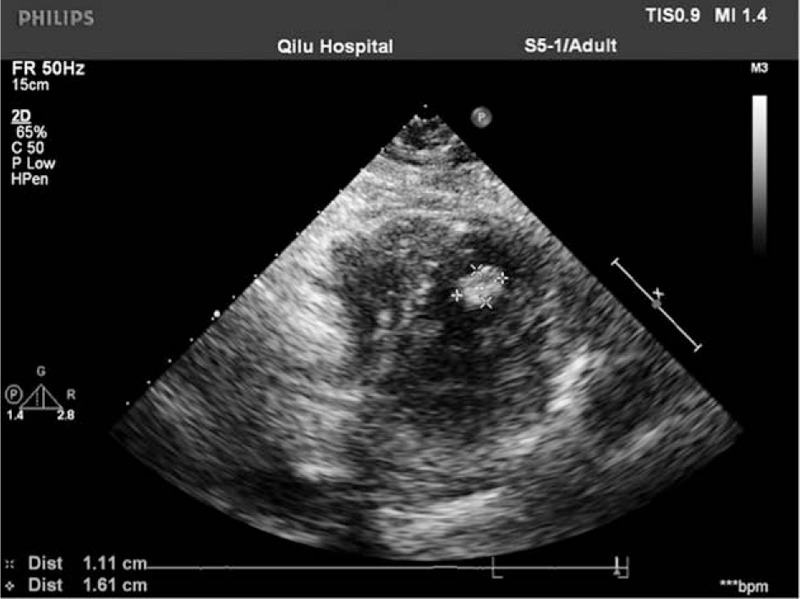
A preoperative transthoracic echocardiogram showed a hyperechoic mass in the left ventricle.

A 70-year-old woman was admitted to the hospital because of fainting and general malaise for 5 years, worse in the prior 6 months. The symptoms were mainly triggered by flexing the neck and changing body position. There was no other discomfort and no significant past history. Her body mass index was 28.52, with blood pressure 144/77 mm Hg, and a radial pulse rate of 80 beats/min and regular. Physical examination showed slight pitting edema in both legs. She had no pathologic cardiac murmur or significant abdominal findings. The echocardiogram revealed a LV mass attached to the posterior wall near the apex, measuring 16.1 mm × 11.1 mm (Fig. 1). The mass had a well-defined border and moved when the heart contracted. LV function and outflow were not impaired. There was no associated thrombus or mass in any other chamber or on any valve. Laboratory tests, electrocardiography, coronary angiography, and brain magnetic resonance imaging (MRI) showed no abnormalities.

**Figure 2:**
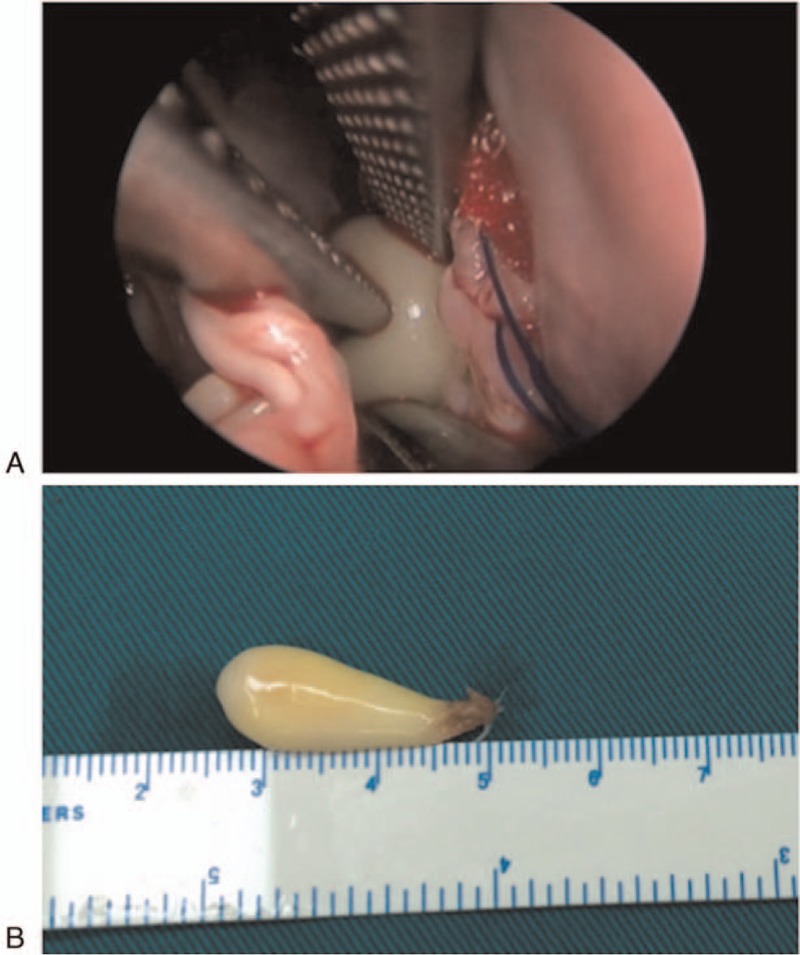
The yellow well-encapsulated left ventricular tumor was revealed clearly by a thoracoscope, and fully resected in the surgery.

**Figure 3:**
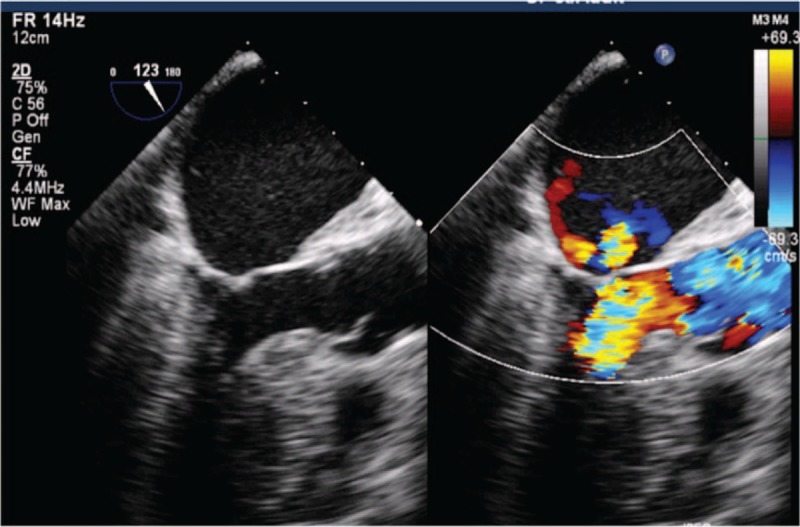
The postoperative transesophageal echocardiogram showed that the LV tumor was resected completely.

Surgery was performed through a limited median sternotomy with cardiopulmonary bypass. An intraoperative transesophageal echocardiogram confirmed that the echodense mass was attached by a pedicle to the LV posterior wall adjacent to the apex. As valve motion prevented a clear surgical view because the deeply located mass was near the apex, we inserted a thoracoscope into the ventricle to help visualize the tumor. The mass was found to be a lipoma, based on its well-encapsulated, yellow appearance. The tumor (25 mm × 10 mm) was carefully removed using scissors and suction (Figs. 2 and 3). No invasion in the ventricular muscle was observed.

**Figure 4:**
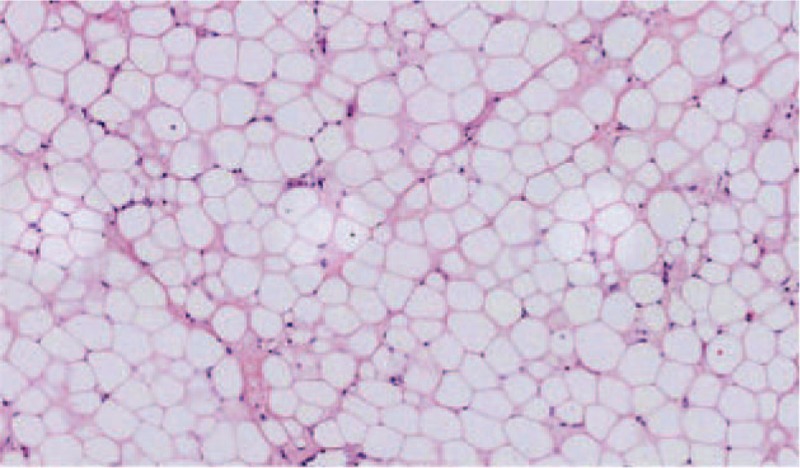
The postoperative pathological result showed that mature fat cells were filled in the tumor, with no immature lipoblasts or heteromorphic cells. (Hematoxylin-eosin staining, 200×).

Histopathologic examination was consistent with a lipoma (Fig. 4). Postoperative recovery was uneventful and the patient was charged 20 days after surgery. No signs of recurrence were detected on an echocardiogram during a 3-month follow-up period (data not shown).

## 3 Literature review and discussion

Primary cardiac tumors can be classified into different types on the basis of their tissues of origin, and include myxomas, fibromas, lipomas, and rhabdomyomas. Myxomas account for over 50% of cardiac tumors, and lipomas only about 8.5% to 20%. A lipoma in the left ventricle is especially rare. A comprehensive review of the literature revealed a total of 20 cases of LV lipoma. Patient data and tumor characteristics are summarized in Table 1.^[4–22]^

**Table 1:**
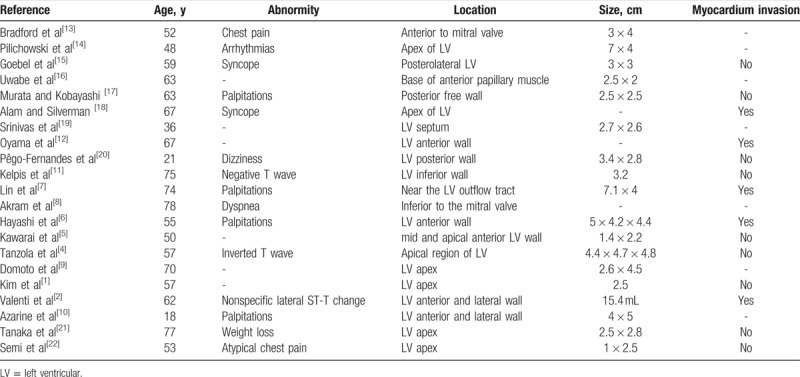
Documented cases of left ventricular lipoma based on PubMed search.

LV lipoma is a benign cardiac tumor, with equal incidence rates in patients of different ages and both sexes.^[23]^ Some grow slowly and may be asymptomatic in the early stage. Therefore, early diagnosis is often missed unless routine or further cardiac examinations are performed for other diseases.^[24]^ The electrocardiogram may show nonspecific ST-T changes.^[2,4,5]^ As it grows, the LV lipoma occupies a larger space, with possible serious consequences through an effect on adjacent cardiac structures or obstruction of the LV cavity. At that time, clinical symptoms such as cardiac murmurs, arrhythmias, fainting, or palpitations^[6]^ may be caused by obstruction of the LV inflow or outflow tract, impairment of cardiac valve function, and involvement of conductive tissue.^[7,8]^ Even if discomfort is present, LV lipoma may be initially misdiagnosed as another cardiac disease because of the nonspecific symptoms. In our case, the patient complained of repeated fainting and malaise for 5 years, but craniocerebral and blood vessel examination showed no relevant pathology. In the months before presentation, symptoms indicating cardiac insufficiency appeared, and an echocardiogram revealed the LV tumor.

The diagnosis of LV lipoma based on history, auscultation, and X-ray alone is difficult. Before the widespread use of echocardiography, some LV tumors were discovered on cardiac catheterization, but most were found at autopsy.^[1,9]^ The echocardiogram is currently preferred for diagnosis of LV lipoma, and can directly and noninvasively observe tumor size, shape, location, activity, relationship with adjacent tissues, and influence on cardiac diastolic function. In addition, cardiac spiral computed tomography, radionuclide scanning, MRI, and especially fat-suppression MRI can show tumor density and blood supply, and further help in the diagnosis.^[10,11]^ Our preoperative diagnosis was myxoma, but postoperative pathology showed a lipoma, suggesting that careful preoperative qualitative diagnosis is necessary.

There has been no report of LV lipoma detachment that caused an embolism. However, as the left ventricle is the most important blood-pumping structure, most researchers believe that LV space-occupying lesions should be resected as early as possible, and that surgery is the only effective treatment. Surgery for LV lipoma in the early stage can reduce the complications of surgery and reduce the death rate. In this case, the patient was placed on cardiopulmonary bypass. The operation was performed through a limited median sternotomy, with an intraoperative transesophageal echocardiogram helping monitor the tumor. During surgery, we found that the tumor was so close to the cardiac apex that we inserted a thoracoscope into the ventricle to help with exposure. The well-encapsulated, yellow tumor was fully resected.

LV lipomas may or may not invade the myocardium. The former may be dangerous. In 2 cases with myocardial involvement, the LV lipoma was deeply invasive and close to the left anterior descending artery and its septal branches. Therefore, en bloc resection was difficult and approximately one-fourth of the tumor was left unresected. In such cases, regular postoperative follow-up is especially crucial. In our patient, postoperative follow-up showed significant symptom improvement and no sign of recurrence.

Postoperative pathological examination is necessary for definitive diagnosis of LV lipoma. Most lipomas are composed of mature, differentiated fatty tissue, with small amounts of fibrous connective tissue and blood vessels, and are completely surrounded by a fibrous membrane.

LV lipoma resection has certain risks. In 2002, a Chinese case reported a death. In that case, LV function had already been impaired before surgery because of the huge volume of the lipoma (8 cm × 8 cm × 4 cm).^[23]^ The 34-year-old patient developed refractory ventricular fibrillation and LV failure within 14 hours after surgery and finally died. Two cases of LV lipoma were treated with conservative therapy, aimed at improving cardiac function.^[12,24]^ Both had a good outcome. Thus, for LV lipoma, diagnosis at an early stage and individualized treatment are essential.

## Author contributions

**Conceptualization:** Wenyu Sun.

**Methodology:** Guangyi Liu.

**Software:** Xiangfei Sun, Guangyi Liu.

**Supervision:** Hwahwi Kim, Wenyu Sun.

**Writing – original draft:** Xiangfei Sun, Hwahwi Kim.

**Writing – review & editing:** Hwahwi Kim, Wenyu Sun.
